# Does art reduce pain and stress? A registered report protocol of investigating autonomic and endocrine markers of music, visual art, and multimodal aesthetic experience

**DOI:** 10.1371/journal.pone.0266545

**Published:** 2022-04-14

**Authors:** Anna Fekete, Rosa M. Maidhof, Eva Specker, Urs M. Nater, Helmut Leder

**Affiliations:** 1 Department of Cognition, Emotion, and Methods in Psychology, Faculty of Psychology, University of Vienna, Vienna, Austria; 2 Department of Clinical and Health Psychology, Faculty of Psychology, University of Vienna, Vienna, Austria; 3 Vienna Cognitive Science Hub, University of Vienna, Vienna, Austria; University of Pisa, ITALY

## Abstract

The pain- and stress-reducing effects of music are well-known, but the effects of visual art, and the combination of these two, are much less investigated. We aim to (1) investigate the pain- and (2) stress-reducing effects of multimodal (music + visual art) aesthetic experience as we expect this to have stronger effects than a single modal aesthetic experience (music/ visual art), and in an exploratory manner, (3) investigate the underlying mechanisms of aesthetic experience, and the (4) individual differences. In a repeated-measures design (music, visual art, multimodal aesthetic experience, control) participants bring self-selected “movingly beautiful” visual artworks and pieces of music to the lab, where pain and stress are induced by the cold pressor test. Activity of the pain and stress responsive systems are measured by subjective reports, autonomic (electrocardiography, electrodermal activity, salivary alpha-amylase) and endocrine markers (salivary cortisol).

## Introduction

In the 2020’s Oscar nominated documentary The Cave [1] which focuses on an underground hospital in Syria, Doctor Salim plays classical music to his patients during surgery telling them: “Don’t worry dear, we don’t have anaesthesia, but we have music!”

But can art reduce pain? And if so, can only music achieve this effect or also other forms of art? In this study we aim to understand if art can reduce pain (and stress) in order to assess if art can be an effective tool for pain and stress management.

Not only is pain one of the biggest global health problems, but its prevalence is continuously increasing as the population ages [2]. Today, pain serves as a leading reason for seeking medical care [3]. Because of the unpleasant sensory and emotional nature of pain, and the associated tissue damage [4], there is a great interest to reduce the noxious sensory and affective components of pain [5] with accessible tools in daily life.

Beside pain, stress is another global health problem [6]. The maladaptive responses of stress [7] result in bad mood, reduced sense of well-being, and negative health outcomes [8]. Given that chronic stress facilitates the development of morbidity [9], tools that can reduce stress in daily life are of great societal interest.

Pain and stress are closely intertwined. Pain is often associated with activity in stress-responsive systems like subjective acute stress, autonomic and endocrine activity [10], and can be influenced by these [11]. Therefore, concomitant investigation of both pain and stress is essential.

Art can be a powerful, and—in its digitized form—an accessible resource, creating a cost-effective tool to promote health in everyday life [12, 13]. The health-related benefits of art have gained more and more attention in the last two decades as evidenced by the recent report of the WHO, reviewing literature from 2000 to 2019, on the role of art in improving health and wellbeing [13]. This report indicates that engaging with (e.g. arts and crafts activity) and participating in (e.g. attending cultural events) the arts—performing arts, visual arts, design, literature, cultural events, digital art—can be a useful tool for both physical and mental health through the components of aesthetic engagement, active imagination, sensory activation, cognitive and emotional content [14, 15], and in some cases social interactions, physical activity, health thematic coping, or interaction with healthcare. Through these components, the effects of appreciating an artwork provide a multifaceted tool that can be used to target outcome variables on a psychological, physiological, social and behavioural level. Aesthetic experience can also be associated with the experience of aesthetic chills, a feeling of shivering, coming along with emotional and psychophysiological reactions [16]. Although other components of art can be considered effective, here we focus on beauty as the most important component. Beauty is a psychological sensation perceived as a sensory impression; this sensation is pleasant, positive [17] and meaningful, often delightful or enjoyable. Psychologically, however, beauty is also a distinctly subjective, often even private, feeling that reflects one’s own experiences in a special way [18].

In this paper, our aims are the following: first, we aim to investigate the multimodal effects of aesthetic experience on pain, and second, on stress. Specifically, we are interested in the effects of modality (music vs. visual art and their combination). Within the frame of these aims, we will also investigate whether music can potentially influence pain and stress more than visual art. Third, in an exploratory manner, we aim to investigate underlying mechanisms of aesthetic experience. Fourth, in an exploratory manner, we aim to investigate how individual differences play a role in pain and stress perception. Below, we first review research showing that art (music and visual art) can reduce pain and stress. Subsequently, we discuss how art can do this. Second, we discuss similarities between music and visual art in order to then discuss why multimodal aesthetic experiences (combining music with visual art) may be more beneficial than single modal experiences. Third, we will discuss the underlying mechanisms of aesthetic experience and the relevant individual differences that might contribute to more effective pain and stress reduction to some people.

### Art, pain, and stress

#### Music

Music, which is used and investigated more often in pain management than other forms of art [19], can be effective both in the context of a multimodal pain management program [20] and when conducted alone [21]. With regard to the quality of music, the importance of beauty in music-based tools for the improvement of health has been emphasized [22, 23]. However, even though different studies showed pain-reducing effects of pleasant (e.g., [24]) and preferred (e.g. [25–27]) music, the effects of explicitly beautiful music on pain have not yet been investigated in well-controlled studies. Beauty, however, is more than just pleasing harmony [23] since aesthetic experience integrates multidimensional psychological mechanisms [14]. A meta-analysis [21] reported that listening to music decreases pain in terms of psychological aspects of subjective pain sensation and emotional distress, furthermore physiological aspects like heart rate, blood pressure, and respiration rate decreased, and listening to music resulted in a smaller amount of analgesic intake.

Music is also commonly used in stress management [28, 29]. Similar to pain, mere music listening can also reduce stress parameters [e.g. 30–33]. Also comparable with pain, the effects of beauty in music on stress are not yet investigated in controlled studies even though beautiful music is recommended for clinical use [22, 23]. De Witte et al. [34] in their systematic review and two meta-analyses showed that listening to music influences subjective stress levels, as well as stress-responsive systems of the body like the autonomic nervous system and the hypothalamus pituitary axis. In sum, music can reduce both pain and stress on a psychological as well as a physiological level.

#### Visual art

When it comes to visual art as a tool for pain reduction, findings are mixed. Only one study found that beautiful paintings decreased pain perception through positive attentional distraction exhibited by pain-related cortical responses [35]. Other findings regarding the analgesic effects of visual art are controversial: for example, compared with self-selected music, visual art has not been found to influence pain tolerance nor perceived control [36].

The stress alleviating effects of viewing visual art are mixed [37] and were mainly investigated in museum and gallery settings. Gallery visits caused faster recovery from high stress assessed by salivary cortisol level [38], and having an aesthetic experience in a religious cultural heritage site resulted in a decrease of 60% salivary cortisol level [39] compared to the normally associated level of cortisol decrease during the circadian cycle (as importantly, these two studies [38, 39] had no other control groups). Furthermore, artworks—especially figurative ones in comparison with modern art—decreased systolic blood pressure as an indicator of stress relief [40]. However, results are not consistent: recovery from stress was found to be slower when viewing landscape paintings in comparison with viewing scrambled images as a control [41]. In addition, stress relieving effects of a single visual art episode [42] have not yet been tested in a controlled lab environment. In the lab setting, other confounding factors such as the physical context of the museum or gallery [43] or social influence [44] with its potential negative impact [45] could be avoided.

In summary, the effects of art on pain and stress are mixed. While music has been shown as an effective tool in both pain and stress management, the same cannot be said about visual art. Therefore, it is important to look at the underlying mechanisms—how can art influence pain and stress—in order to understand why music is an effective tool, as well as to investigate if visual (or other modalities of) art have the potential to be equally effective.

### How can art influence pain and stress?

**Fig 1:**
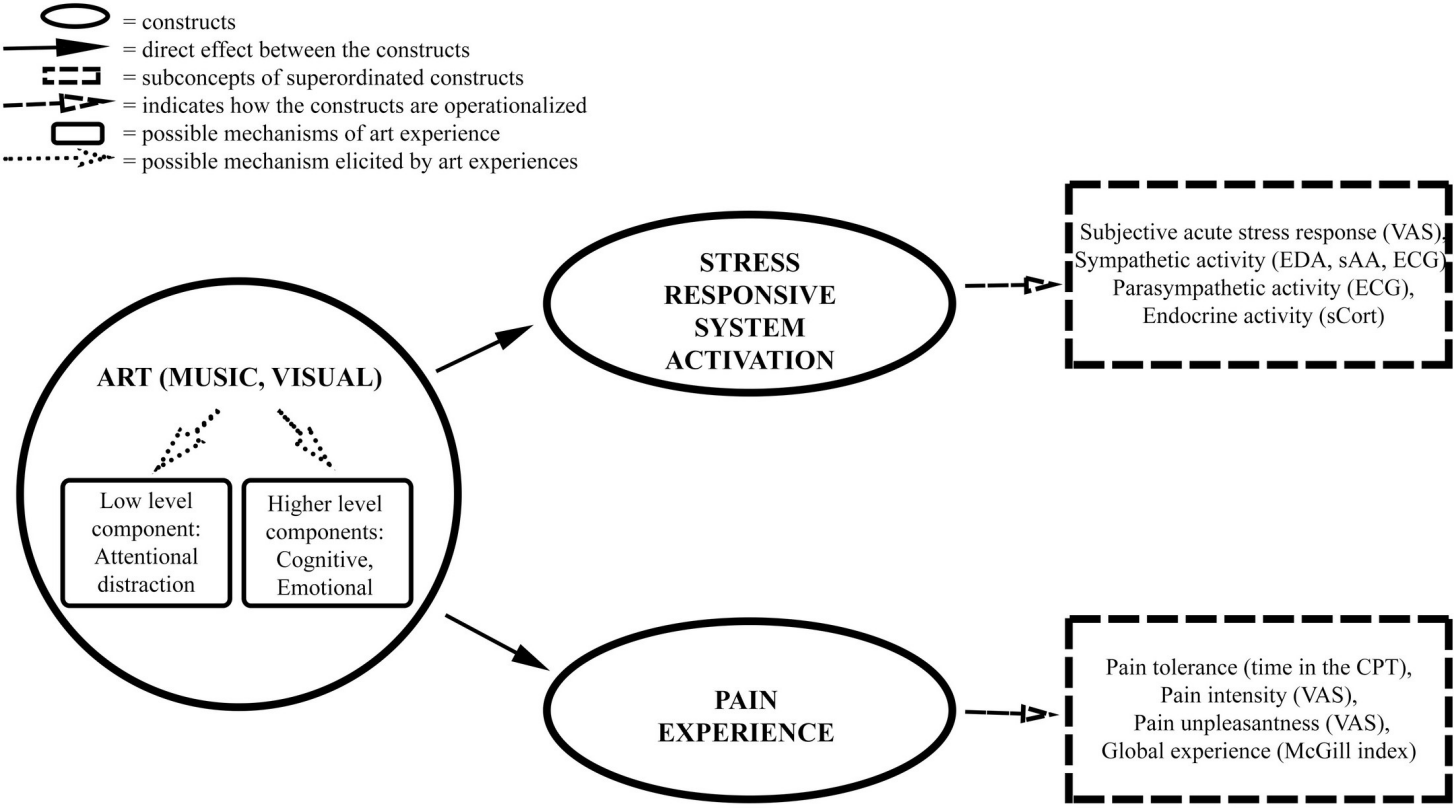
Proposed mechanisms on how art can influence pain and stress. CPT = Cold Pressor Test, ECG = electrocardiogram; EDA = electrodermal activity; sAA = salivary alpha-amylase; sCort = salivary cortisol, VAS = Visual Analogue Scale.

The perception of both music and visual art includes aesthetic experiences that share pleasing and rewarding neural [46] and psychological mechanisms [47, 48], therefore both music and visual art are expected to be beneficial regarding individuals’ well-being through appreciation of beauty [49]. In the following, we outline how aesthetic experiences in music and visual art can influence pain and stress-responsive systems through both neural and psychological mechanisms (Fig 1).

### How can art influence pain?

Listening to music during pain induction modulates activity regions of brain, brainstem, and spinal cord indicative of descending pain modulation [50]. Similarly, beautiful visual art can influence the activity of different cortical areas that activate pain-modulating structures in the brainstem [35]. These constant neural processes are associated with psychological mechanisms.

Psychological mechanisms of pain reduction by listening to music were recently reviewed and classified into categories by Howlin and Rooney [51]. The mechanisms they propose (details below) are similar to models of visual aesthetic experience [15, 42, 52], encompassing first sensory-perceptual, and later cognitive and affective evaluations. Given this similarity in the underlying processes, it would theoretically be possible for visual art to be equally effective (in terms of reducing pain and stress) to music. Below, we give a detailed overview of the mechanisms proposed for music, and at each step discuss what the visual art equivalent would be.

Firstly, low level attentional resources are allocated to the music away from pain experience, which leads to a distraction from experienced pain [51]. Similarly, visual art is able to modify pain through attentional distraction [35]. Thus, both music and visual art have the potential to reduce pain by redirecting attention away to the aesthetic experience.

Secondly, higher level cognitive and emotional components have been considered as analgesic mechanisms. Through the use of music, active and focused listening, encompassing cognitive mechanisms of extra-musical associations, memories or visual imagery [53] and affective mechanisms, reduces pain [51]. Through the use of visual art, affective modulation of pain is possible [5, 54, 55] since aesthetic experience serves as a powerful tool to counter pain experience through the wide range of complex emotions of pleasure, contemplation, and amazement [56]. Similarly, aesthetic experience can be associated with emotional arousal, liking of the aesthetic stimulus [57] and a wandering mind [58], and these concepts can in turn be associated with pain perception [36, 59, 60]. Thus, both music and visual art have the potential to reduce pain by engaging thoughts and evoking emotions [61]. An important agent in this context is personal meaning which is considered as a core mechanism of pain-modulation through music listening and strengthens the sense of self [36, 62]. Through listening to music, energizing, encouraging, and motivational effects result in renewed personhood that leads to physical well-being, and this effect is made stronger with self-selected music [63]. This is in line with the finding that most of the time self-selected music was found to be more effective in music interventions than pieces chosen by others [20]. Through a visual aesthetic experience, high level of self-relevance that later results in metacognitive self-awareness leads to powerful, transformative aesthetic experiences described by feeling of “cathartic” release, epiphany, enlightenment, and harmony [15, 64]. Thus, both music and visual art have the potential to reduce pain more effectively through self-relevant processes.

### How can art influence stress?

Similar mechanisms on neural and psychological levels can also explain the impact of arts on stress-responsive systems. Music can modulate activity in neuronal networks including amygdala, nucleus accumbens, and ventral tegmental area [65] that are associated with the regulation of hormonal and autonomic responses to rewarding and emotional stimuli [28]. Consistently, various studies show changes in hormonal and autonomic responses as well as subjective stress levels in response to music [30–34, 65]. Similarly, it seems plausible that beautiful visual artworks can modulate stress levels since activity in the amygdala has been found for the perception of visual art [48]. Thus, both music and visual art have the potential to reduce stress by modulating attentional as well as higher order cognitive and emotional processes.

An overview of the underlying mechanisms is given in Fig 1. From our discussion, it seems evident that both music and visual art have the potential to reduce pain and stress, however, due to the similarity of the underlying processes, the question arises if a combination of music and visual art (multimodal experience) has the potential to be even more effective than music or art on its own (single modal experience). Furthermore, experiences of pain and stress are multimodal in nature. For example, pain is not only a sensory experience but also an affectively laden experience. Therefore, a multimodal tool (in our case—aesthetic experience) that aims to reduce pain and stress may be more effective than a single modal tool. We discuss this line of thinking in more detail below.

### Multimodal experience of aesthetics, pain, and stress

As noted, the experience of pain has multidimensional components on the sensory domain (intensity) and on the affective domain (unpleasantness) [5]. These are closely intertwined and cannot be selectively alternated [66], therefore tools targeting different dimensions of pain are considered beneficial—aesthetic experience, which is associated with attentional, cognitive and emotional processes, can be such a tool.

Similarly, stress affects changes on a neural, physiological, behavioural, cognitive, and emotional level [67], again, therefore, a multifaceted approach is considered to be beneficial. These overlapping components of pain and stress can be addressed through the corresponding attentional, cognitive and emotional mechanisms of aesthetic experience.

Aesthetic experience comprising of multiple modalities—with perceptual, sensory, affective, and cognitive components [15, 42, 52]—is assumed to have the potential to provide a more effective tool in the context of pain and stress management than the single modalities. Multimodal aesthetic experience is assumed to be efficient through multimodal integration of attentional, cognitive and emotional aspects, and through a cross-modal transfer through the different sensory domains.

Multisensory stimulation through different senses—e.g., hearing and seeing emotional content—supports each other’s effects through multimodal integration [68]. Since the literature shows increasing support for the assumption of shared attentional resources between the modalities of hearing and vision [69], it can be assumed that multimodal integration might be associated with higher attentional distraction for multimodal aesthetic experience. Also, higher level mechanisms can be assumed to be stronger activated in multimodal aesthetic experience than in the single modalities. For example, targeting a wider range of emotions through music and visual art could serve as a more powerful tool than each modality on their own. In detail, multimodal aesthetic experience can be beneficial since the quality of involved emotional responses partly differs: listening to music elicits more intense vitality-related emotions like feelings of tenderness, nostalgia, peacefulness, power, joy and potential sadness [70]. In contrast, viewing paintings compared to listening to music elicits more intense wonder and reduced vitality of emotions [57, 70]. Besides the quality of involved emotions, subjective complexity has a multidimensional nature regarding music and visual stimuli [71]. Its relation to hedonic value (liking, beauty, pleasantness) also differs between music and visual art: in music, the relationship between complexity and beauty followed an inverted-U curve, while in paintings, complexity was positively associated with beauty [71, 72]. In summary, lower level and higher-level processes associated with music and visual art are assumed to support each other through multimodal integration of aesthetic experience.

Besides multimodal integration, multimodal aesthetic experience is also assumed to be beneficial through cross-modal transfer of different sensory domains: arousal induced by listening to Romantic piano music altered the arousal felt when looking at pictures, but the music did not transfer pleasantness [73]. On the other hand, listening to music can promote beauty judgements as it has been found that listening to nineteenth-century music resulted in evaluating faces more attractive due to misattribution of arousal [74]. As the two domains of music and visual art can alter each other, investigating aesthetic experience across various sensory domains is crucial [75]. Regarding the interplay of music and visual art, aesthetic experiences with different modalities of films, music, and visual arts were found to be described with general terms of beautiful, wonderful, and original, even though more emotionally loaded words were used for music and films than visual art [76]. In addition, aesthetic appreciation was found to be higher when people were exposed to congruent music and visual artworks: they enjoyed abstract paintings of Kandinsky more when listening to abstract music of Anton Webern, and enjoyed impressionists paintings of Monet more when listening to impressionist music of Ravel [77]. Similarly, aesthetic experience was enhanced for figurative art with classical music listening, meanwhile abstract art was enjoyed more with jazz music [78]. More importantly, Actis-Grosso et al. [78] showed that paintings are experienced as more pleasant in multimodal conditions (i.e. with music) in comparison to silence conditions.

To conclude, a combination of different modalities of aesthetic experience might be an enhanced stimulation and interplay of attentional, cognitive and emotional processes that influence the reactions from pain and stress stimuli. In order to accurately assess how aesthetic experience may reduce pain and stress, different forms of aesthetic experience need to be disentangled—studied separately and in combination. It is of high importance to further investigate modality-specific characteristics of the underlying mechanisms and to integrate them in a common framework from a multimodal perspective [79]. In order to find out if the underlying mechanisms of aesthetic experience are activated for different forms of aesthetic experience, attentional, cognitive and emotional aspects need to be investigated.

### Role of individual differences

Pain-reducing effects through music listening have been found to be stronger for persons with higher level of trait empathy, measured by the participants’ tendency to transpose themselves imaginatively into the feelings and actions of fictitious characters [80]. The authors explain this by emotional engagement with the music, which is assumed to be higher for persons with high level in trait empathy, resulting in a more absorbing activity. In line with this, absorption (i.e., engagement) with music has been suggested to modulate pain experience [81]. Also, the effects of music listening on stress-responsive systems can be modulated by empathy [82]. Additionally, absorption is a promising modulator of stress-reactions to music listening [83, 84]. Similar pain- and stress-reducing effects of absorption by means of visual art have not been found yet, however we argue that visual art might have this potential, too, as it has been found that when people were viewing visual art installation, their absorption increased as a function of how much awe they experience [85].

Similarly, empathy [86] and absorption [87] are also associated with emotional engagement in the context of viewing visual art. Even though, to our knowledge, the role of trait empathy and absorption has not yet been investigated in the context of pain- and stress-reducing effects of visual art, it can be assumed that enhanced emotional engagement through trait empathy and absorption will influence pain and stress-responsive systems also for the use of visual art, since underlying emotional mechanisms are assumed to be similar for the two modalities of art, as explained above.

### Present study

In the present study, we aim to—for the first time—test systematically: (1) the effect of multimodal (music, visual, and combined) aesthetic experience on pain and (2) stress-responsive systems, and in both cases, compare the single modalities (e.g., testing whether music or visual art is more effective); in an exploratory manner, (3) investigate the underlying mechanisms of aesthetic experience; and (4) the individual differences.

To our knowledge, only one study has attempted to investigate the effects of different modalities of aesthetic experience—visual art and music—on pain perception induced by a cold pressor test (CPT) [36]. However, this study has several important limitations. Firstly, though Mitchell et al. [36] included both music and visual art, they only investigated single modal experience, and did not assess the potential benefits of multimodal aesthetic experiences on pain experience. We aim to go further by looking at both modalities separately and in combination.

Secondly, participants could not bring their self-selected visual artwork to the lab—as they could their preferred music. In our study, we aim for consistency in the choice of the stimuli by letting participants bring both self-selected music, and self-selected visual artworks. This was necessary so that participants maintained personal meaning with their chosen stimuli—as elaborated above.

Finally, Mitchell et al. [36] only looked at pain and did not investigate the stress-responsive systems. As discussed above it is necessary to investigate pain and activity of stress-responsive systems together in order to better understand the underlying mechanisms. We address this in our study by investigating the association between pain and stress during aesthetic experience. For the investigation of the underlying mechanisms of aesthetic experience, we will investigate the activation of attentional, cognitive and emotional aspects. In order to account for individual differences, the role of trait empathy and absorption will be investigated.

The benefits of this research are multifaceted. For the first time, the effects of the single modalities of music and visual art on pain and stress responses are systematically compared with each other and with multimodal (music+ visual art) aesthetic experiences. By investigating the underlying mechanisms of aesthetic experience, our findings provide insight into which facets of aesthetic experience (attentional, cognitive and emotional processes) influence which correlates of pain and stress experience the most. Therefore, future art interventions will be able to target these most influential facets and thus become more effective. By investigating personality differences, individually tailored art interventions will be possible. As our approach combines clinical psychology and empirical aesthetics, our findings will contribute to the improvement and development of individualized pain and stress management programs through modalities of art, and therefore improve well-being of society in general.

### Hypotheses

Detailed hypotheses for each combination of dependent and independent measures can be found in Table 1 in S1 File that provides a summary of research questions, hypotheses, analysis plan and interpretations. Broadly speaking we will test two confirmatory hypotheses:

1. We hypothesize that (1.1) multimodal (music + visual art) aesthetic experience will reduce pain more than single modal experience; and that (1.2) music will reduce pain more than visual art.
2. We hypothesize that (2.1) multimodal (music + visual art) aesthetic experience will reduce activity in stress-responsive systems more than single modal experience; and that (2.2) music will reduce activity in stress-responsive systems more than visual art.

In an exploratory manner, we aim to instigate (3) the lower (attentional) and higher (cognitive and emotional) processes of potential underlying mechanisms on how multimodal aesthetic experience influences pain and stress experience. Furthermore, we explore (4) the potential individual differences—specifically trait empathy and trait absorption—that might influence the effects of aesthetic experience on pain and stress.

## Materials and methods

### Design

**Fig 2:**
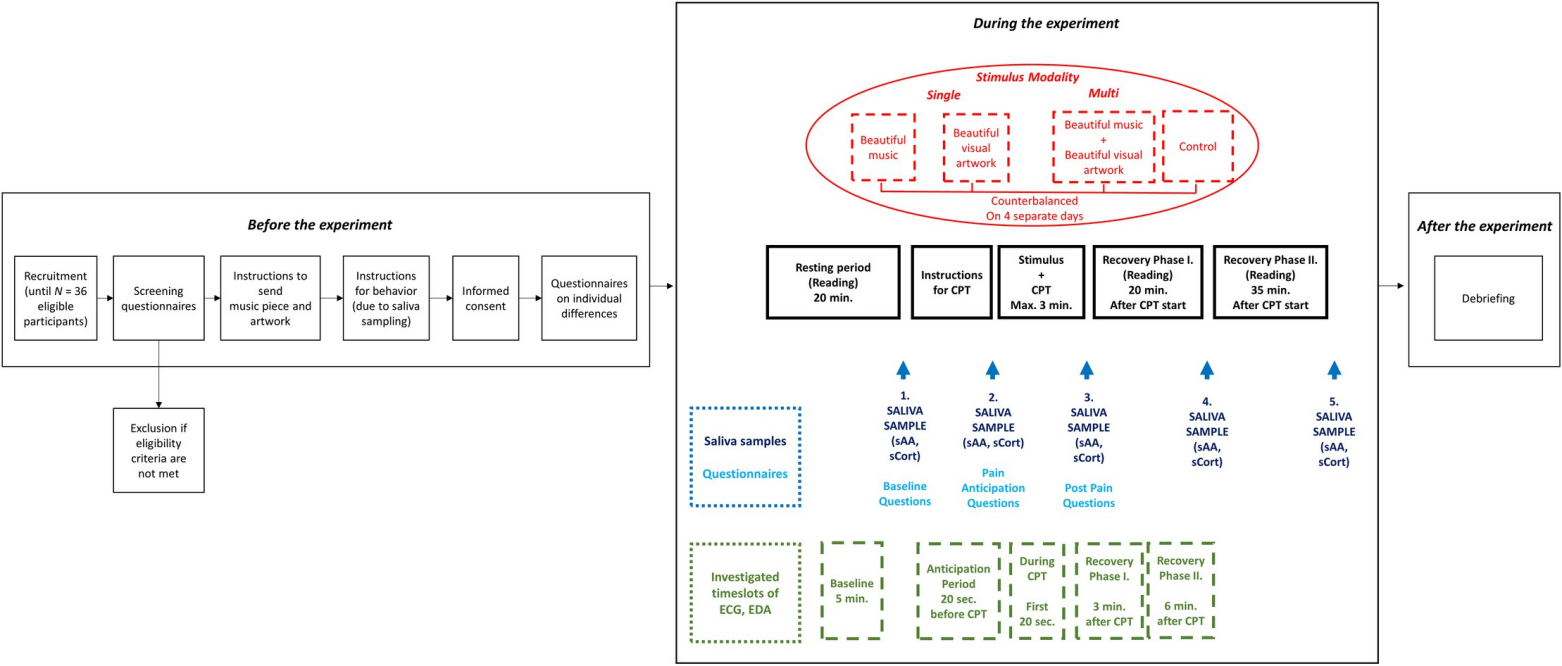
Procedure of the study (before, during, after the experiment). CPT = cold pressor test; ECG = electrocardiogram; EDA = electrodermal activity; sAA = salivary alpha-amylase; sCort = salivary cortisol.

We will use a within-subjects design with four conditions: music, visual, multimodal, and control (see Fig 2 for the conditions). A within-subjects design was chosen because this was found to be the most suitable for pain research due to the wide range of significant individual differences that occur in response to pain [88]. However, this comes with the need to have sufficient length of washout period between the trials and prevent carry-over effects. To ensure that these conditions are met, each of the 4 conditions will be administered during a separate session on a different day (at least 24 hours between sessions since we expect this elapsed time is sufficient to prevent carryover effects, however, this timeframe is exploratory since currently no research study is available on this regard). Thus, participants will need to come to the lab 4 times (1 condition per session) on 4 different days. The order of conditions will be randomized (with random number generator) and counterbalanced. We will use Latin square to counterbalance the sequences of the conditions (with 4 conditions, 4! = 24 possible sequences, therefore these 24 sequences will be repeated 1.5 times since we calculated a sample of 36 participants). Testing time will always be in the afternoon (between 11:30 am and 5:00 pm), since it has been found that pain perception was lowest during the mornings if pain is induced by cold [89] and because of the diurnal changes in cortisol [90].

### Participants

Sample size was calculated with G*Power 3 [91] by using repeated measures ANOVA by providing four groups of participants (see hypothesis 1 for the main investigation of our primary outcome variables), hence the four measurements. Medium effect size of Cohen’s *f* of .23 was used since this effect size has been found for the effect of self-selected music stimuli on pain intensity reduction in adult sample of a *Cochrane* meta-analysis and review [92] (Note that Cepeda et al. [92] reports an overall effect size (mean difference) of -.46 that we transformed to Cohen’s *f* of .23 because that was needed for G*Power sample size calculation). The power analysis indicated a total sample size of 36 participants to achieve a power of .90 with an α of .05, while the correlation among repeated measures was assumed to be of 0.5. Since drop-out rates may be high (participants have to complete 4 separate sessions), we intend to collect data until we have complete data (i.e., 4 completed sessions) for 36 participants. Participants are recruited through the participant database system of the University of Vienna, via flyers, and social media. The online platform offers a description of the study, informs participants about the eligibility criteria and they can register to their preferred dates and timeslots for the study. Interested persons are then contacted by the experimenter and are checked for eligibility criteria with detailed online screening questionnaires. Participants are included and excluded based on standard practice that have been used in e.g. [30, 31].

### Eligibility criteria

Participants are included if they are between the age of 18 and 29 years (Emerging Adulthood [93]), have an average Body Mass Index (BMI) from 18.5 to 25 kg/m², speak German fluently, and in case of women have regular menstruation. Participants are excluded from the study if they have colour blindness, inappropriate visual acuity, hearing problems, art or music-related profession, or art- or music-related studies, mental disorders, cardiovascular diseases, arterial occlusive diseases, very high or very low blood pressure, chronic pain, diabetes, Raynaud’s syndrome, epilepsy, or a recent serious injury. Further exclusion criteria include regular intake of pain-reducing medication, or medication intake with impact on stress-responsive systems, smoking more than five cigarettes per week and current drug consumption. Additional exclusion criteria of female participants include: momentary pregnancy or breastfeeding, premenstrual syndrome, use of hormonal contraceptives.

We are aware that the strict eligibility criteria bring the limitation of generalization of the findings. However, because of the novelty of the study, it is necessary to make sure that we do not risk participants’ safety (see also e.g. McIntyre et al. [94] and exclude participants with characteristics that influence the perception of the presented stimuli (see also e.g. Madsen and Moore [95] or our outcome variables (see also e.g. Adam and Kumari [96]; Carr and Goudas [97]. Therefore, we only test those persons who meet these eligibility criteria, also based on related literature (e.g. Linnemann, Ditzen, et al. [30]; Linnemann, Strahler et al. [31])

Participants will receive course credits (16 credits) or monetary compensation (40€) for participation in all sessions. Partial compensation of 2 credits/5€ is granted after each of the first three sessions, and 10 credits/25€ is given after attendance of the last session. The study has already been approved by the ethics committee of the University of Vienna (reference number: 00506) and will be carried out in accordance with the Declaration of Helsinki.

### Materials

#### Stimuli

Participants will be asked to provide self-selected pieces of music and artworks that they find “movingly beautiful” (see also [98, 99]) because they reliably elicit beauty and pleasure [100]. In the music and visual artwork conditions, the stimulus is presented as single modality.

The participants send a digital reproduction of their self-selected “movingly beautiful” piece of music or visual artwork, via e-mail prior to the experiment. To ensure congruency, we ask participants to select music and visual art that are a good fit together.

In case of self-selected music, the pieces should be no longer than seven minutes and no shorter than three minutes. The maximum length aims that the main content of the music piece falls into the CPT exposure (e.g., if the music piece is much longer, it is possible that the first three minutes are an introductory part and the most powerful part of the piece comes later when the CPT is over), whereas the minimum length of the music piece reflects the length of the CPT (3 minutes). The piece of music should contain no breaks or silences, meaning that participants should not be exposed to silence any time during the CPT. The selected recording of the piece of music should have a good sound quality. The pieces of music will be displayed with active acoustic noise cancelling JBL LIVE 460NC headphones.

With the self-selected visual artworks, the images are required to have a minimum resolution of 720×576 pixels.

The visual artworks are presented with a flexible code that adjusts the presentation size depending on the resolution of the image to be presented the best way on an LCD screen of 30 inches, with a capacity of 2560x1600 pixels resolution. This process is used to ensure perceived control and fulfil the self-selected criteria in the movingly-beautiful music/visual artwork condition.

In the multimodal condition, the self-selected music piece and the self-selected artwork are displayed at the same time, again during the pain induction. In the control condition, participants are instructed to wear noise-cancelling headphones with no auditory stimuli display and view the grey screen during pain induction.

#### Cold pressor test

We intend to use the CPT since it was found to reliably and effectively elicit pain experience [101, 102]. The CPT was found to be applicable to induce stress response [103], for example by increasing HPA axis activity, resulting in an increased cortisol concentration [104], as well as by increasing sympathetic nervous system activity, resulting in increased skin conductance [105] and elevated blood pressure [104].

Participants are asked to immerse their dominant hand—since the non-dominant hand is equipped with EDA sensors [106]—to a circulating cold water bath and to leave it there until it is too uncomfortable to continue. They are asked to immerse the hand until the wrist, not to form a fist with their fingers, and to not touch the container. Crushed ice is used to make sure that the water remains cold for the duration of the test, and the ice is covered with a metal grid to prevent contact. The temperature of the water is kept constant at 1°C (acceptance range: >0.5°C and <1.5°C) and monitored by a digital probe thermometer (ATP Messtechnik GmbH Ettenheim, resolution = 0.1°C, accuracy = ± 0.5°C), and the water is constantly circulated with a pump in order to avoid laminar warming around the submerged hand. During the CPT, the stimulus of one condition is presented with the same onset of the stimuli and hand immersion. The maximum duration of the CPT is 3 minutes.

### Measures

#### Pain measures

Pain tolerance is measured in time [ms] once per condition. The experimenter stands behind the participant with a portable keyboard, and when the participant submerges their hand into the cold water, the experimenter presses the onset button which activates the visual stimulus—appearing on the screen/ the music stimulus—playing the music through headphones. When the participant removes the hand from the cold water, the experimenter presses the button again, indicating the offset of the CPT, and the end of the stimulus presentation.

Global pain perception is measured by the McGill Pain Questionnaire [107, 108] after the CPT (see also Table 3 in S1 File), furthermore pain intensity, pain affect and perceived control are measured five times per condition with Visual Analogue Scales (VAS; [109])—a continuous line on which participants can rate their perception with 0 (e.g. meaning in the VAS of pain intensity: no pain at all) and 100 (e.g. meaning in the VAS of pain intensity: most intense pain that can be imagined) as end points.

#### Stress measures

For the assessment of the subjective acute stress level, the item “I feel stressed” will be rated with a VAS ranging from 0 (not at all stressed) to 100 (maximally stressed). The use of a single VAS for the assessment of subjective acute stress has been validated in a clinical context [110] and is also widely used in other studies in related research fields (e.g. Feneberg et al., [111]; Thoma et al. [32]).

*Physiological measures of stress*. In order to quantify the ANS activity, electrocardiogram (ECG) is used for the investigation of heart rate variability (HRV) which is quantified in terms of RMSSD (i.e. square root of the mean squared differences of successive heartbeat intervals) as an indicator for parasympathetic activity [112]. Heart rate (HR) is calculated in terms of beats per minute (BPM) as an indicator for both sympathetic and parasympathetic activity. Electrodermal activity (EDA) is assessed by using skin conductance level (SCL) as a parameter for sympathetic activity [113]. Room temperature is held constant between 23 and 24°C to ensure stable EDA.

ECG and EDA data is measured by 8-channel bioamplifier (Mobi 8 BP, TMS International, Enschede, the Netherlands) with a 24-bit A/D conversion rate. Physiological data is sampled at 256 Hz and continuously stored on a hard drive. The amplifier is connected to a separate computer and recorded in MATLAB 9.8 (MathWorks, Inc, USA). ECG active electrode is placed on the left collarbone, meanwhile reference and ground electrodes are placed on the left and right mastoid bones. EDA is measured by two electrodes which are placed on middle and index fingers of the non-dominant hand [106]. The acquisition of unfiltered raw skin conductance data was guaranteed using a custom-specific skin conductance sensor. Before electrode application, the skin is cleaned using curd soap. ECG and EDA parameters are constantly measured but only certain time slots are investigated (a baseline interval of 5 minutes during the initial rest period, 20 seconds immediately before pain induction to indicate anticipation to the CPT, the first 20 seconds during the CPT—those participants who attend the CPT shorter than 20 s are excluded from ECG and EDA analysis because quality of calculated physiological parameters cannot be ensured—, 3 minutes directly after the CPT as a first part of the recovery period, and the subsequent recovery period of 3 minutes).

*Salivary stress biomarkers*. Before coming to the given session, participants are asked to follow instructions in order to reduce confounding factors that can affect salivary alpha-amylase and cortisol. The instructions are sent via mail 24 hours before the session to those participants who met the inclusion criteria. These instructions are standard procedure and based on related literature [114]. Participants are instructed not come to the lab if they feel sick; not to eat in the previous one hour before the session starts; not to use chewing gum within the previous 24 hours before the session starts; not to brush their teeth in the previous one hour before the session starts; not to smoke, not to drink caffeine-containing beverages (coffee, tea, coke), juice, alcohol in the previous 18 hours; not to engage in physical activity in the previous 24 hours; and not to drink water 10 minutes before the sampling. (If a participant did not follow one part of the instructions (we ask participants to fill out a questionnaire at each session to check if they followed the instructions), we make a note, and ask them to follow this practice for each upcoming session (e.g., if someone had a coffee in the morning before the session in the afternoon, then we ask this participant to follow this practice for the upcoming sessions in order to have the same treatment within the participant. We only exclude participants if they feel sick or we see them drinking a coffee and eating something, because this will mean that they did consume food/caffeine in the last hour before the saliva sampling.)

When the participant arrives, we ask them to drink a glass of water in order to remove potential aliment remains and to better standardize the mouth moisture, and emphasize that they can only drink water up to 10 minutes before the sampling.

In each of the 4 sessions, five saliva samples will be collected by means of SaliCaps (IBL, Hamburg, Germany), resulting in 20 samples in total per person. Saliva samples are obtained by collecting the saliva after two minutes without swallowing into a tube by means of a straw. Immediately after collection, samples are stored at– 20°C until analysis. In order to gain information on endocrine activity, salivary cortisol (sCort) is investigated, using a commercial luminescence immunosorbent assay (LUM; IBL, Hamburg, Germany). Also, salivary alpha-amylase (sAA) is investigated, an enzyme used as an indicator of the activity of the sympathetic nervous system [115]. Therefore, a kinetic colorimetric test with reagents from DiaSys Diagnostics Systems (Holzheim, Germany) is applied.

#### Mechanisms of aesthetic experience

In order to get insight which mechanisms are the most influential when art influences pain and stress, we conduct measurements in all conditions: music, visual art, multimodal, control. First, we measure lower level, attentional processes, we ask participants to evaluate their level of *Distraction* that is measured with item: “I was distracted from the cold water by the music and visual artwork/ music/ visual artwork/ the grey screen and the headphones.”.

Regarding the higher level, we measure *Mind wandering* “The [stimulus] made me think about other things, such as memories and emotions.

Furthermore, we measure, in an exploratory way, *Pleasantness* “I found the [stimuli] emotionally positive”; and *Emotional Arousal*: “I found the [stimuli] emotionally arousing”; *Liking* “I liked the [stimuli].”; and complex emotions: “The [stimulus] has evoked the following emotions: joy, sadness, relaxation, anger, fear, nostalgia, melancholy.” *Personal meaning* was measured with “I found the [stimuli] personally relevant.” Aesthetic experience as a whole is measured by *Beauty* with the following items: “I found the [stimuli] ‘movingly beautiful’ in this laboratory environment”; by *Chills* (after an explanation of the term “chills”): “I experienced chills while being exposed to the [stimuli].”; and by *Enjoyment “*I enjoyed [looking at/ listening to the stimulus].” In the multimodal condition only, we measure *Congruency*: “I found that the music piece and visual artwork fit well together.” All items have to be rated on a 5-point Likert scale (from “not at all” to “very much”).

#### Individual differences

We will investigate an indicator of trait empathy with the Questionnaire of Cognitive and Affective Empathy (QCAE; [116]. Items can be answered on a 4-point scale ranging from 1 (= “strong agreement”) to 4 (= “strong disagreement”).

Trait absorption was measured the German version of the Tellegen Absorption Scale (TAS; [117]; based on the original English version by [118]. The questionnaire consists of 34 items that can be answered on a 5-point rating scale.

### Procedure

After the screening procedure for the testing of eligibility criteria (see above), participants will complete questionnaires on trait empathy and trait absorption. Subsequently, they are contacted to send artworks and music piece. Once they have sent their self-selected stimuli set, they are instructed to follow instructions regarding the saliva sampling.

Subsequently, participants will come to the lab 4 times. Each session will be conducted in a quiet lab environment and each session follows an identical procedure (see Fig 2). Initially, participants are seated in a comfortable chair, approximately 1m in front of the monitor, and their non-dominant hand is equipped with devices of electrocardiography (ECG) and electrodermal activity (EDA) to ensure the continuous measurement of ECG and EDA during the whole session. During the resting period of 20 minutes—because the HPA axis has a reaction time of about 20 minutes for cortisol secretion [119]—participants will read natural science journals. Afterward, the first saliva sample is taken as a baseline measurement in order to investigate salivary alpha-amylase (sAA) and salivary cortisol (sCort). Then, participants complete a pre-test consisting of questionnaires on pain and subjective stress levels. Subsequently, participants will be informed that a CPT would follow to induce pain and stress. Immediately before the CPT, in an anticipatory period, participants are to give a second saliva sample and complete questionnaires on pain perception and subjective stress levels. This is followed by the CPT at the same time with the stimulus exposure. After the CPT, participants give a third saliva sample and complete a post-test consisting of questionnaires on perceived pain, stress, and perception of the stimuli. During the subsequent recovery period, participants give two more saliva samples (fourth saliva sample 20 minutes after CPT start, and fifth saliva samples 35 minutes after CPT start) and complete questionnaires on subjective pain and stress levels at both time points during the recovery period. Each session will last for about 70 minutes.

### Statistical analyses

To test hypotheses 1 and 2, two-factor repeated-measures ANOVAs will be conducted in SPSS (version 24.0; IBM Corporation, Armonk, NY, USA). Each dependent primary pain variable will be investigated five times per condition (apart from pain tolerance and Global McGill Pain Index that will be investigated only once per condition, see also Table 3 in S1 File). However, only two time points are of interest for our main hypotheses: baseline measurement and measurement directly after the CPT (Post CPT). Apart from pain tolerance, a difference variable (global McGill pain index: Recovery–Post CPT; other parameters: Post CPT–Baseline) will be calculated for each dependent primary pain variable. Regarding our secondary outcomes (stress parameters), also five time points (or time slots for ECG and EDA parameters) will be investigated. Similarly, to pain, only two time points (or time slots) are of interest for our main hypotheses. Difference variables will be calculated (see also Table 1 in S1 File). *Condition* is included as a factor with four levels (music, visual art, multimodal aesthetic experience, control).

Values of *p* < .05 will be considered as significant in all analyses. For all analyses, *Sex* (male/female) will be added as a covariate.

For more details see Table 1 in S1 File. Summary of research questions, hypotheses, analysis plan and interpretation, and our OSF page (anonymized for the review process): https://osf.io/yxgrv/?view_only=e6f43838c25f4cb889b3a7624060120d.

## Supporting information

S1 File (DOCX)
