# Supplementary material for: Does art reduce pain and stress? A registered report protocol of investigating autonomic and endocrine markers of music, visual art, and multimodal aesthetic experience
Source: PLoS One. 2022 Apr 14;17(4):e0266545. doi: 10.1371/journal.pone.0266545 (PMC9009611; doi:10.1371/journal.pone.0266545)
Supplement: S1 File — (DOCX) [file pone.0266545.s001.docx]

**Does art reduce pain and stress?**

A registered report protocol of investigating autonomic and endocrine markers of music, visual art, and multimodal aesthetic experience

**Table 1. Summary of research questions, hypotheses, analysis plan and interpretation.**

| Question | Dependent Variable | Hypothesis | Analysis Plan | Interpretation given different outcomes |
| --- | --- | --- | --- | --- |
| 1.1. Does multimodal aesthetic experience (music + visual art) influence pain more than the single modalities?  1.2. Does musical aesthetic experience influence pain more than visual art? | Pain  (Tolerance, intensity, unpleasantness, global experience) | 1. 1. Multimodal aesthetic experience  1.1.1. increases pain tolerance,  1.1.2. decreases  a) pain intensity,  b) unpleasantness,  c) global McGill pain index  more than single modal aesthetic experience and control condition.  1.2. Music  1.2.1. increases pain tolerance,  1.2.2. decreases  a) pain intensity,  b) unpleasantness,  c) global McGill pain index  more than visual art and control condition. | One separate repeated measures ANOVA for each dependent variable will be calculated. For each dependent pain variable, one value will be investigated during the experimental task (tolerance in seconds, the other variables are reported retrospectively directly after the CPT).  *Condition* is included as a factor with four levels (music, visual art, multimodal, control).  If the repeated measures ANOVAs show significant effects, post-hoc tests using the Bonferroni correction are conducted to reveal between which conditions the significant difference has been found. | 1.1.1. If the multimodal condition shows higher pain tolerance (*p*≤ 0.05) to one or more other conditions, we will conclude that multimodal aesthetic experience increases pain tolerance.  Otherwise, we will conclude that it does not.  1.1.2. If the multimodal condition shows lower a) pain intensity, b) unpleasantness, c) global pain experience (*p*≤ 0.05) to one or more other conditions, we will conclude that multimodal aesthetic experience decreases that respective aspect (a, b, c) of pain experience. Otherwise, we will conclude that it does not influence one (or all included) aspects of pain experience.  1.2. Similarly, if music shows more  1.2.1 increased pain tolerance and  1.2.2. more decreased a) b) c) than visual art and the control condition, we conclude that music is more effective in influencing pain experience than visual art and the control condition. Otherwise, we will conclude that music is not more effective. |
| 2. 1. Does multimodal aesthetic experience (music + visual art) influence stress more than the single modalities?  2.2. Does musical aesthetic experience influence stress more than visual art? | Stress  (Subjective response; ECG; EDA; sAA; sCort) | 2.1.1. Multimodal aesthetic experience decreases stress. Specifically, we expect a decrease in:  a) *perceived subjective stress* (self-report: it refers to during the CPT attendance, which is retrospectively assessed directly after the CPT)  b) *sympathetic activity* (EDA measures in terms of SCL, sAA and HR)  c) *endocrine activity* (sCort)  2.1.2. Multimodal aesthetic experience increases:  d) *parasympathetic activity* (results in a decrease in HR and an increase in RMSSD)  during a pain test compared to single modal aesthetic experiences and control condition.  (In this case, all measures are compared to a Baseline value (see Fig 2- During the experiement)  2.2. Similarly, we expect that music  2.2.1. decreases stress more than visual art and a control condition, on a), b), c) aspects and  2.2.2. increases d) aspect.  (In case of ECG and EDA, we calculate difference variables (20 seconds during CPT– Baseline value) that we use as a dependent variable.  (Those participants who attend the CPT shorter than 20 s are excluded from ECG and EDA analysis because quality of calculated physiological parameters cannot be ensured.) | One separate repeated measures ANOVA for each dependent variable will be calculated. *Condition* is included as a factor with four levels (music, visual art, multimodal, control).  If the repeated measures ANOVA shows significant effects, post-hoc tests using the Bonferroni correction are conducted to reveal between which conditions the significant difference has been found. | 2.1. If the multimodal condition  2.1.1. a) decreases perceived subjective stress, b) sympathetic activity, c) endocrine activity,  2.1.2. d) increases parasympathetic autonomic activity (*p*≤ 0.05) compared to one or more other conditions, we will conclude that multimodal aesthetic experience influences that respective aspect (a, b, c, or d) of stress experience.  Otherwise we will conclude that it does not influence one (or all included) aspects of stress experience.  2.2. Similarly, if music  2.2.1. decreases stress on a), b), c) and  2.2.2. increases d) aspect more  than visual art and the control condition, we conclude that music influences that respective aspect (a, b, c, or d) of stress experience.  Otherwise, we will conclude that music is not more effective. |

ECG = electrocardiogram; HR = heart rate; HRV = heart rate variability; RMSSD = square root of the mean squared differences between successive heartbeat intervals; EDA = electrodermal activity; SCL = skin conductance level; sAA = salivary alpha-amylase; sCort = salivary cortisol.

**Supplementary Fig 1. Theory and its measurements levels.**


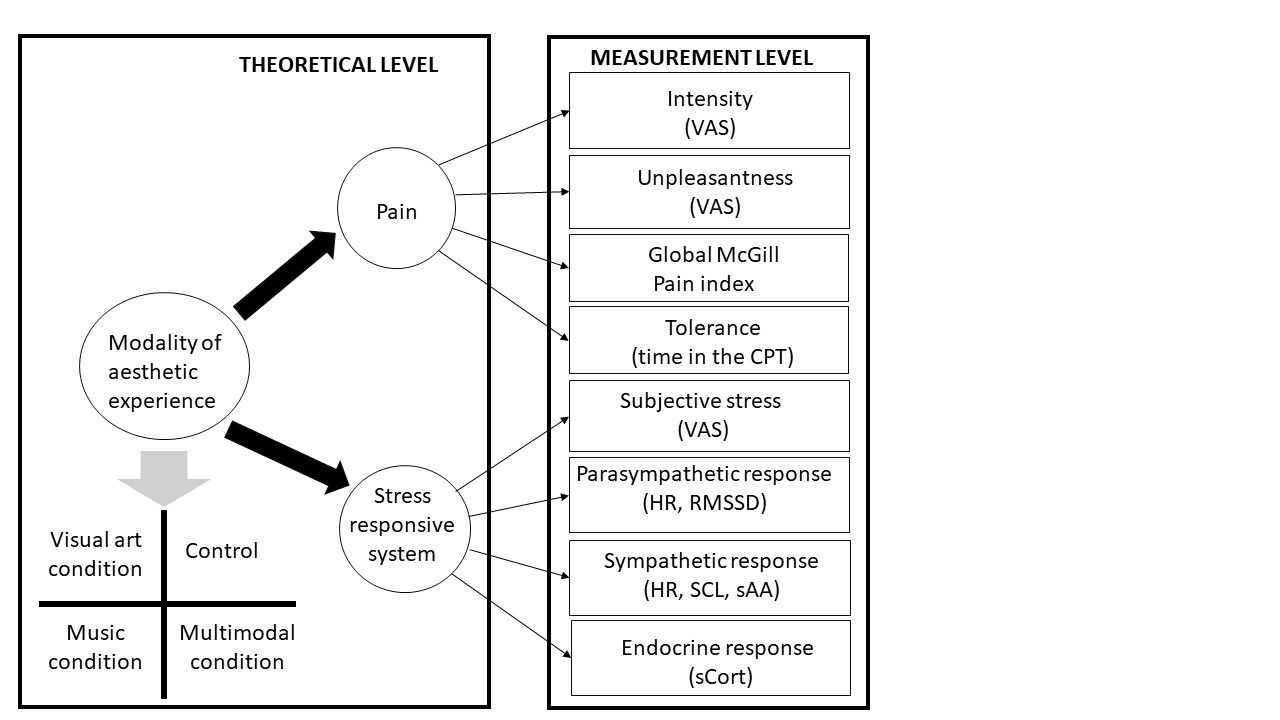


**Table 2. Assessments in the whole course of the study.**

| Construct | Measure | Short name | Online survey previous to the study | Testing day 1 | Testing day 2 | Testing day 3 | Testing day 4 |
| --- | --- | --- | --- | --- | --- | --- | --- |
| *Primary variables* |  |  |  |  |  |  |  |
| Pain tolerance | Time [ms] |  |  | x | x | x | x |
| Pain intensity | Global McGill Pain Index  Visual Analogue Scale pain intensity | VAS pain intensity |  | x  x | x  x | x  x | x  x |
| Pain affect | Visual Analogue Scale pain unpleasantness | VAS pain unpleasantness |  | x | x | x | x |
| *Secondary variables* |  |  |  |  |  |  |  |
| Acute subjective stress | Visual Analogue Scale stress | VAS stress |  | x | x | x | x |
| ANS activity | ECG parameters |  |  | x | x | x | x |
|  | EDA parameters |  |  | x | x | x | x |
|  | sAA |  |  | x | x | x | x |
| Endocrine activity | sCort |  |  | x | x | x | x |
| Stimuli-related perceptions for the assessment of psychological mechanisms of aesthetic experience | Stimuli-related perceptions |  |  | x | x | x | x |
| Trait empathy | Questionnaire of Cognitive and Affective Empathy | QCAE | x |  |  |  |  |
| Trait absorption | Tellegen Absorption Scale | TAS | x |  |  |  |  |
| *Variables for description or checking of eligibility* |  |  |  |  |  |  |  |
| Demographic data | Questions on demographic data |  | x |  |  |  |  |
| Informed consent |  |  | x | x |  |  |  |
| Depression | Beck Depression Inventory | BDI-II | x |  |  |  |  |
| Menstrual cycle | Questions on menstrual cycle |  | x |  |  |  |  |
| Mental health | Patient health questionnaire | PHQ-D | x |  |  |  |  |
| Premenstrual syndrome | Premenstrual Syndrome Questionnaire | PMS questionnaire | x |  |  |  |  |
| Retrospective personal view on the study | Post-monitoring items (open-ended question regarding subjective experience) |  |  |  |  |  | x  (right after completing session 4) |

Short form of the McGill Pain Questionnaire (SF-MPQ-D) [1,2]; Beck Depression Inventory (BDI-II; [3]; Patient health questionnaire (PHQ-D;[4]; Premenstrual Syndrome Questionnaire (PMS questionnaire; [5]; German version of the Questionnaire of Cognitive and Affective Empathy (QCAE; [6]; Tellegen Absorption Scale (TAS; [7]; based on the original English version by [8].

**Table 3. Assessments during one testing day (one session).**

| Construct | Measure | Short name | During resting period | Directly after resting period | Directly before the CPT | During the CPT | Directly after the CPT | Recovery 1  (20 min after CPT start) | Recovery 2  (35 min after CPT start) |
| --- | --- | --- | --- | --- | --- | --- | --- | --- | --- |
| *Primary variables* |  |  | | | | | | | |
| Pain tolerance | Time [ms] |  |  |  |  | x |  |  |  |
| Pain intensity | Global McGill Pain Index  Visual Analogue Scale pain intensity | VAS pain intensity |  | x | x |  | x  x | x | x |
| Pain affect | Visual Analogue Scale pain affect | VAS pain affect |  | x | x |  | x | x | x |
| *Secondary variables* |  |  | | | | | | | |
| Acute subjective stress | Visual Analogue Scale stress | VAS stress |  | x | x |  | x | x | x |
| ANS activity | ECG parameters |  | x | x | x | x | x | x | x |
|  | EDA parameters |  | x | x | x | x | x | x | x |
|  | sAA |  |  | x | x |  | x | x | x |
| Endocrine activity | sCort |  |  | x | x |  | x | x | x |
| Stimuli-related perceptions | Stimuli-related perceptions |  |  |  |  |  | x |  |  |

Short form of the McGill Pain Questionnaire (SF-MPQ-D) [1,2]; ECG = electrocardiogram; EDA = electrodermal activity; sAA = salivary alpha-amylase; sCort = salivary cortisol.

**References**

1. Melzack R. The McGill Pain Questionnaire: Major properties and scoring methods. Pain. 1975;1: 277–299. doi:10.1016/0304-3959(75)90044-5

2. Oesch P, Eberhardt R, Hilfiker R, Keller S, Kool J, Luomajoki H, et al. Assessments in der Rehabilitation - Band 2: Bewegungsapparat. Hogrefe Verlag; 2017.

3. Hautzinger M, Keller F, Kuehner C. Beck Depressions-Inventar Revision (BDI-II) Manual. 2009.

4. Löwe B, Spitzer R, Zipfel S, Herzog W. PHQ-D: Gesundheitsfragebogen für Patienten; Manual Komplettversion und Kurzform. 2002.

5. Ditzen B, Nussbeck F, Drobnjak S, Spörri C, Wüest D, Ehlert U. Validierung eines deutschsprachigen DSM-IV-TR basierten Fragebogens zum prämenstruellen Syndrom. Z Klin Psychol Psychother. 2011;40: 149–159. doi:10.1026/1616-3443/a000095

6. Reniers RLEP, Corcoran R, Drake R, Shryane NM, Völlm BA. The QCAE: A questionnaire of cognitive and affective empathy. J Pers Assess. 2011;93: 84–95. doi:10.1080/00223891.2010.528484

7. Ritz T, Dahme B. Die Absorption-Skala: Konzeptuelle Aspekte, psychometrische Kennwerte und Dimensionalität einer deutschsprachigen Adaptation. Diagnostica. 1995;41: 53-61. Available: https://psycnet.apa.org/record/1995-87269-001

8. Tellegen A, Atkinson G. Openness to absorbing and self-altering experiences (“absorption”), a trait related to hypnotic susceptibility. J Abnorm Psychol. 1974;83: 268–277. doi:10.1037/H0036681
